# The ProVent model learns to speak French

**DOI:** 10.1186/s13054-014-0576-z

**Published:** 2014-10-20

**Authors:** Christopher E Cox

**Affiliations:** Department of Medicine, Duke University School of Medicine, DUMC 102043, Durham, NC 27710 USA

## Abstract

Leroy and colleagues report on the accuracy of the Prolonged Mechanical Ventilation Prognostic Model (‘ProVent’) in a cohort study of patients ventilated for at least 21 days in one of three hospitals in the north of France. This study is noteworthy because it is the first to describe the performance of the ProVent model both outside the US and in a community hospital-based setting.

## Commentary

Prolonged mechanical ventilation is an international phenomenon [1]. Prolonged mechanical ventilation, or ‘chronic critical illness’, is defined by at least 10 days of mechanical ventilation [2]. Patients who receive prolonged mechanical ventilation are important to consider because they face high mortality, experience significant physical and emotional morbidity, and incur high costs to health-care systems [3]. Decision making for their family members and clinicians alike is often difficult because of the uncertainties associated with their outcomes.

For these reasons, Carson and colleagues [4] developed the Prolonged Mechanical Ventilation Prognostic Model (‘ProVent Score’) for 1-year mortality a few years ago before this patient population was well appreciated by many clinicians. ProVent is important for a number of reasons. First, clinicians clearly need help to improve their sense of prognosis because their accuracy for long-term outcomes prediction is poor for this group. Few intensive care unit clinicians actually provide longitudinal care and are simply unaware that outcomes can be so poor for those who might have survived an acute illness but recovered insufficiently to be quickly liberated from a ventilator. Second, ProVent requires only five variables (each adding 1 point to a total ProVent score) yet is quite accurate across a variety of medical and surgical diagnoses. This simplicity lends itself to actual clinical use rather than relegation to research application only. These variables can be linked within electronic health records to inform clinical care because they are objective and easy to extract. And research purists may be heartened by the existence of a ProVent model with beta estimates that inform a regression model. Third, ProVent contributed to interventions developed to improve communication and decision making for family members of these patients. To date, this tool has been validated in US populations with simple clinical data obtained at 10, 14, and 21 days of ventilation.

Yet the article by Leroy and colleagues in the previous issue of *Critical Care* is important in its own right [1]. These authors first of all describe a patient population that is less well characterized in Europe than in the US, where the long-term care industry emerged in part to provide their post-hospital care - a health-care sector that is even publicized in the lay press [5,6]. Although it is true that medical care delivery varies substantially across continental Europe, many European patients still receive prolonged mechanical ventilation at great costs, the purported US-European differences in management and decision-making style notwithstanding [7]. And it is reasonable for one to suspect that this population may grow further given many European centers’ notable enthusiasm for high-intensity therapies such as extracorporeal life support.

Especially noteworthy about the study by Leroy and colleagues is its execution in three hospitals in the north of France, near the Belgian border. This community-based research venue stands in contrast to the academic medical center setting in which the original ProVent model was developed. Criminally few studies are performed in non-tertiary care academic settings. This status quo serves to perpetuate stereotypes: academics may think community physicians are not current, while community physicians may wonder whether academic medical center trial populations truly reflect their patients. Contributing to the rich history of French critical care research, the authors have provided a unique view of community-based critical care research that hopefully will stimulate others to do the same.

In the first study of the ProVent 21-day model conducted both in a community-based sample and outside the US, these authors found that this score accurately identified patients at high risk of death - receiver operator characteristic of 0.74 (95% confidence interval 0.67 to 0.81); a plot of the true-positive rate versus the false-positive rate - with a notably clear separation of subgroups arranged by progressive model points. They also describe a novel iteration of ProVent (‘French ProVent’) that includes only three variables (age of at least 65 years, requirement for hemodialysis, and use of vasopressors) yet had a receiver operator characteristic (0.74) similar to that of the standard five-variable version. One could perhaps dismiss this innovation as a simpler version of an already simple model, though it may possess advantages when applied within constrained databases. Finally, the authors show evidence for the universality of prolonged mechanical ventilation outcomes - patients’ general long-term experiences are sobering, whether care is provided in North Carolina or in Nord-Pas-de-Calais.

There are still some very interesting questions that involve prolonged mechanical ventilation and ProVent. How exactly do we as clinicians apply this information to clinical care and communication, and is it different between the US and Europe, as some have suggested? Can the model be broadened successfully to include outcomes that patients and families value, such as long-term cognitive function, physical independence, and quality of life?

ProVent seems to be one of the uncommon models that holds up well outside its place of origin. Given the model’s success in these French hospitals, the critical care community would be well served by further investigations of ProVent in other large study databases and national health records from other European and Asian nations where chronic critical illness is common. Such a common language of understanding what to expect from the long-term outcomes of chronic critical illness early in the course would be welcome. However, for now, ProVent is served well by learning a new language.
